# Scalp Acupuncture Attenuates Brain Damage After Intracerebral Hemorrhage Through Enhanced Mitophagy and Reduced Apoptosis in Rats

**DOI:** 10.3389/fnagi.2021.718631

**Published:** 2021-12-20

**Authors:** Peng Liu, Xinyang Yu, Xiaohong Dai, Wei Zou, Xueping Yu, Mingming Niu, Qiuxin Chen, Wei Teng, Ying Kong, Ruiqiao Guan, Xiaoying Liu

**Affiliations:** ^1^First Affiliated Hospital, Heilongjiang University of Chinese Medicine, Harbin, China; ^2^Clinical Key Laboratory of Integrated Traditional Chinese and Western Medicine, Heilongjiang University of Chinese Medicine, Harbin, China; ^3^Structural Biology and Developmental Neurobiology, St. Jude Children's Research Hospital, Memphis, TN, United States; ^4^Second Affiliated Hospital, Heilongjiang University of Chinese Medicine, Harbin, China; ^5^Integrated Chinese and Western Medicine, Zhongshan Hospital, Fudan University, Shanghai, China

**Keywords:** scalp acupuncture, intracerebral hemorrhage, mitophagy, apoptosis, Baihui, Qubin

## Abstract

To study the effect of scalp acupuncture (SA) on the mitophagy signaling pathway in the caudate nucleus of Sprague-Dawley rats following intracerebral hemorrhage (ICH). An ICH model was established by injecting autologous arterial blood into the caudate nucleus in 200 male Sprague-Dawley rats, which were divided into five groups: sham, ICH, 3-methyladenine group (3-MA, 30 mg/kg), SA, and SA+3-MA. Animals were analyzed at 6 and 24 h as well as at 3 and 7 days. Composite neurological scale score was significantly higher in the SA group than in the ICH group. Transmission electron microscopy showed less structural damage and more autophagic vacuoles within brain in the SA group than in the ICH group. SA group showed higher levels of Beclin1, Parkin, PINK1, NIX protein, and a lower level of Caspase-9 in brain tissue. These animals consequently showed less neural cell apoptosis. Compared with the SA group, however, the neural function score and levels of mitophagy protein in the SA+3-MA group were decreased, neural cell apoptosis was increased with more severe structural damage, which suggested that 3-MA may antagonize the protective effect of SA on brain in rats with ICH. SA may mitigate the neurologic impairment after ICH by enhancing mitophagy and reducing apoptosis.

## Introduction

Intracerebral hemorrhage (ICH) remains the most serious and intractable type of stroke in the world. Approximately two million people are affected each year (Keep et al., 2012), one in three dies within one month of the event (Krishnamurthi et al., 2013). Most patients are left with severe nerve defects and reduced quality of life. Primary brain damage after ICH includes physical damage such as hematoma, which can lead to secondary brain damage that is propagated by mitochondrial dysfunction, activation of microglia, and release of neurotransmitters and inflammatory mediators (Fan et al., 2019). Ultimately, these responses lead to apoptosis and necrosis of the brain tissue, two strong determinants of neurological deterioration and poor prognosis (Bobinger et al., 2018).

Autophagy is involved in ICH pathophysiology (Niu et al., 2017) and is essential for homeostasis, including in the nervous system, but its detailed effects in physiology and pathophysiology are still poorly understood, limiting its usefulness as a therapeutic target. For example, one study showed that inhibiting autophagy helped reduce the severity of brain damage after iron-induced ICH (Chen et al., 2012), whereas another study showed that promoting autophagy decreased early brain injury in subarachnoid hemorrhage (Jing et al., 2012).

Intracerebral hemorrhage disturbs energy metabolism (Liu et al., 2018a) and so may involve a specific form of autophagy called mitophagy, which promotes mitochondrial renewal, maintains mitochondrial homeostasis, and removes damaged mitochondria (Ozden et al., 2011). It is possible that ICH involves the same mitophagy pathways as Parkinson's disease, which is attributed to mitochondrial dysfunction (Nunnari and Suomalainen, 2012). Insights from mitophagy proteins in Parkinson's may provide candidates for research in ICH. In Parkinson's, the PTEN-induced putative kinase protein 1 (PINK1)/PARK2 gene-encoded E3 ubiquitin ligase (Parkin) signaling cascade activates mitophagy (Chang et al., 2017). In hypoxic Parkinson's brain tissue, PINK1 can bind and stabilize the mitochondrial membrane after mitochondrial depolarization. PINK1 recruits the E3 ubiquitin ligase Parkin from the cytosol, and Parkin forms complexes with p62 and histone deacetylase (HDAC)6 to ubiquitinate proteins on the mitochondrial surface. These complexes interact with LC3, promoting formation and recruitment of mitochondrial autophagosomes (Geisler et al., 2010). Beclin1 can also participate in the translocation of Parkin to the mitochondrial membrane, thus promoting its interaction with mitophagy initiating factors (Choubey et al., 2014). NIX/BINP3L is a mitophagic receptor that helps clear mitochondria during mammalian reticulocyte maturation (Schweers et al., 2007), and it can bind to Atg8 autophagy family proteins to initiate mitophagy. NIX can also compete with Beclin1 for binding to Bcl-2, resulting in free Beclin1 that induces autophagy (Maiuri et al., 2007).

Autophagy can be stimulated by scalp acupuncture (SA) after ICH (Liu et al., 2019). SA has been widely used in China for thousands of years and has become a widespread complementary treatment for epilepsy, Parkinson's disease, and stroke (Liao et al., 2012). SA can stimulate the release of several neurochemicals and promote cerebral microcirculation, normal metabolism, neurogenesis, angiogenesis, and neuronal activity (Weng et al., 2016), but the exact mechanism has not yet been elucidated. In this work, we examined whether SA may activate autophagy, specifically mitophagy, after ICH in rats.

## Materials and Methods

### Animals

This study involved 200 male Sprague-Dawley rats (6–7 weeks old, 250 ± 30 g) from the Animal Experiment Center of Heilongjiang University of Chinese Medicine (certificate SCXK(Heilongjiang) 2017-018). Animals were housed in a pathogen-free facility maintained at 45–65% ambient humidity, temperature of 23 ± 2°C, and 12-h day-night cycles. Food and water were provided *ad libitum*.

Prior to experiments, animals were deprived of food and water for 12 h, then they were allocated into the following five groups (*n* = 40 each): sham, ICH, 3-methyladenine (3-MA), SA, and SA+3-MA. These groups were treated as described below. Each group was divided into four subgroups and evaluated at 6 h, 24 h, 3 days, and 7 days after treatment. 3-MA is an autophagy inhibitor that reduces the occurrence of autophagy in subarachnoid hemorrhage without affecting the extent of bleeding (Jing et al., 2012).

### ICH Induction

**Figure 1:**
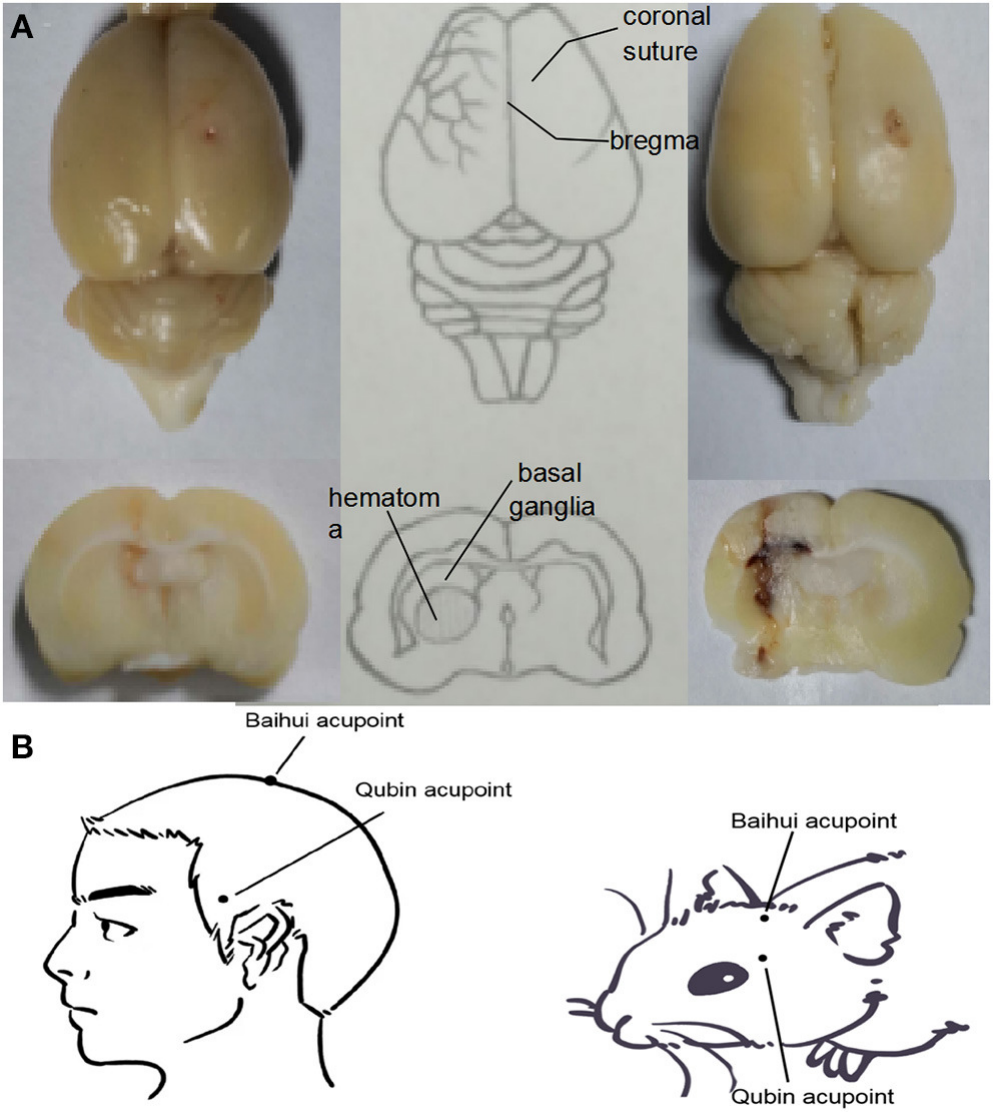
**(A)** Location of the ICH hematoma. Schematic showing the position of the blood transfusion into the caudate putamen. **(B)** Location of Baihui acupoint and Qubin acupoint in human and rats. Baihui acupoint is located at the midpoint of the line between the double ear tips on the parietal bone, and Qubin acupoint is located at 2/3 of the line from the orbital margin to the external ear hole.

Intracerebral hemorrhage was achieved through blood autotransfusion, as described by Hua et al. (2002) in 3-MA, SA, and SA+3-MA groups. Rats were anesthetized intraperitoneally with pentobarbital (34.62 mg/kg, Selleck, Shanghai, China). Animals were immobilized in the prone position using a stereotaxic device (STW-3X, Chengdu Instrument Factory, Chengdu, China) to ensure that the anterior and posterior fontanelles remained on an even plane. After hair removal and disinfection, a 1-cm median incision was made in the scalp. The periosteum was pulled away to expose the bregma and the coronal suture of the cranium. A dental drill was placed 3.5 mm to the right of the bregma and 0.2 mm posterior to it (Figure 1). Then a circular opening with a diameter of 1 mm was bored with a dental drill down to the dura mater surface (Yang et al., 2018). The tail was disinfected, 2 cm was cut off from the end, and blood (50 μL) was collected using a microsyringe. The same microsyringe was inserted through the drill hole to the depth of the caudate putamen (~6 mm). The blood from the tail was injected into the caudate putamen at 25 μL/min, and the needle was maintained in position for ~2 min. Then the microsyringe was slowly removed, the skull opening was sealed with dental cement, and the skin was sutured and sterilized. Rats from the sham group underwent sham surgery without receiving blood, but received an injection of equal dose of saline. In the process of modeling, we placed rats on a thermostatic water blanket to keep their rectal temperature at about 37.5°C. When rats woke up and ate freely, the blanket was removed. According to Bederson' s scale, the model was successful if the score was 1–3 at 2 h after modeling (Bederson et al., 1986).

### SA and 3-MA Treatment

**Figure 2:**
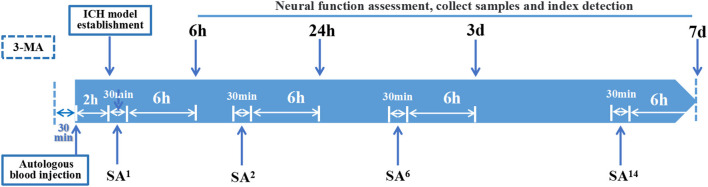
Time axis: At 30 min before establishing ICH model, 3-MA was injected into the lateral ventricle of rats in 3-MA and SA+3-MA group. The success rate of ICH model establishment was evaluated 2 h after autologous blood injection. All established rat models were given acupuncture for the first time. Rats were treated with SA once, twice, 6 times, and 14 times for 6-h, 24-h, 3-day, and 7-day models, respectively. Each treatment takes 30 min. Neurological function, sampling and target detection were evaluated at 6 h, 24 h, 3 days, and 7 days after SA treatment respectively.

At 6 h after ICH surgery, SA and SA+3-MA rats underwent SA intervention twice daily using no. 28 needles (Φ 0.35 × 40 mm, HuaTuo Brand, Suzhou Medical Appliance, Suzhou, China), which penetrated the DU20 (Baihui) acupoint for 1.5 cm through to the GB7 (Qubin) acupoint on the lesion side of the brain. Each treatment session was for 30 min. For each 30-min session, needling at 180–200 rpm was performed for three sessions, each lasting for 5 min (Figure 2). Rats were treated with acupuncture one time, two times, six times, and 14 times for 6 h, 24 h, 3 days, and 7 days, respectively. At 30 min before ICH, 3-MA (20 mg/ml in saline) was injected intracranially at 30 mg/kg (Duan et al., 2017) into 3-MA and SA+3-MA animals.

### Neural Function Assessment

At 6 h after all interventions, neural function was evaluated using the composite neurological scale score described by Garcia (Garcia et al., 1995). This composite contains a battery of tests to examine the following spontaneous activity, symmetry of limb movement, lateral rotation, forelimb movement, axial sensation, tentacle proprioception, and crawling. Animals were scored on a scale from 3 (lowest) to 21 (highest) points. Lower score indicates greater severity of neurological defects.

### Histopathology

At 3 days after ICH, rats were injected intraperitoneally with pentobarbital (60 mg/kg) and then perfused with 4% paraformaldehyde. Brain tissue was removed, stained with hematoxylin for 5 min, destained with 1% hydrochloric acid-ethanol for 5 s, soaked in ammonia for 10 s, and sliced into 4-micron sections. Sections were counter-stained with eosin for 3 min, rinsed, coverslipped, and sealed with neutral gum. Images were captured using a light microscope (BX53; Olympus, Tokyo, Japan) at 400× magnification.

### Transmission Electron Microscopy

Following sedation and perfusion as described for histopathology, brains were collected and placed in 2.5% glutaraldehyde buffer (pH 7.4). Five sections (1 × 1 × 1 mm^3^) were excised from the lateral margin of the hematoma and fixed with 1% osmium. Samples were dehydrated through an ethanol gradient and embedded in epoxy resin. Sections were sliced to a thickness of 0.5 μm with an ultramicrotome (UC6; Leica, Wetzlar, Germany) at room temperature and stained with saturated uranium acetate. Sections were analyzed at 100,000× magnification using a transmission electron microscope (CM 100; Philips, Amsterdam, Netherlands), and digital images were taken using an ORCA-HR camera (Hamamatsu, Shizuoka, Japan).

### Immunohistochemistry

Brain tissue (5 μm) were cut and fixed in formalin buffer after being paraffin-embedded. Paraffin-embedded sections of brain tissue were first deparaffinized and dehydrated. Slides were incubated with 0.3% H_2_O_2_ for 10 min and washed with ddH_2_O three times, 3 min each time. Antigen retrieval was undertaken in a pressure cooker for 10 min with 0.01 M citrate buffer and washed with phosphate-buffered saline (PBS) three times, 5 min each time. Five percent bovine serum albumin (BSA) was applied to the specimen sections to block nonspecific protein for 20 min at room temperature. The primary antibodies, which were rabbit antibodies against Beclin1 (bs-1353R, Bioss, China), Parkin (bs-23687R, Bioss, China), PINK1 (6946, CST, USA), or BNIP3L/NIX (12396, CST, USA), were incubated for 2 h at 37°C and washed with PBS three times, each time 3 min. After washing, slides were washed and then incubated with secondary antibody, goat anti-rabbit IgG (ab7090, Abcam, UK), at 37°C for 30 min and washed with PBS three times, 3 min each time. The sections were stained with diaminobenzidine for ~5–10 min. Five nonoverlapping visual fields at 400× magnification were analyzed from each sample using a Moticam 3000 (Hong Kong Special Administrative Region, China) microphotography system. The mean number of positive cells was calculated using Image-Pro Plus 6.0 software (Xue et al., 2014).

### Terminal Deoxynucleotidyl Transferase (TdT)-Mediated DUTP Nick end Labeling (TUNEL)

Paraffin sections of brain tissue were dewaxed, rehydrated in water, and permeabilized at room temperature for 30 min in 10 mM Tris/HCl (pH 7.4-8.0) containing pepsin K (20 μg/ml). Sections were rinsed two times with phosphate-buffered saline (PBS), incubated with 50 μL TUNEL reaction mixture for 1 h at 37°C, rinsed three times with PBS, incubated with 50 μL of inverter-POD for 30 min at 37°C, and again rinsed three times with PBS. Sections were incubated with DAB at room temperature for 10 min, thoroughly rinsed with distilled water, and counterstained with hematoxylin for 1 min. Coverslips were sealed with neutral gum sealant. Three slices of each rat brain tissue were selected for observation, and five nonoverlapping visual fields around the hematoma were selected for each slice. The images were taken from penumbra region. Visual fields were captured at 400e magnification using the Motic3000 system. Mean positivity was calculated using Image-Pro Plus 6.0.

### Western Blot Assay

We selected the striatum in brain tissue for sampling. The striatum is the prone site of intracerebral hemorrhage. We used a stereotactic instrument to accurately inject autologous blood into the rat brain. We take brain tissue and preserve the tissue with a thickness of about 6 mm centered on the injection point. Brain tissues were homogenized in lysis buffer and centrifuged, and the supernatant (50 g total protein) was fractionated by SDS-PAGE. Proteins were transferred to a polyvinylidene fluoride membrane and blocked in 5% skim milk for one hour at room temperature. Membranes were incubated at 4°C overnight with rabbit primary antibodies against Beclin1 (Bioss, China), Parkin (Bioss, China), PINK1 (CST, USA), BNIP3L (CST, USA), or Caspase-9 (bs-0049R, Bioss, China). Blots were incubated with rabbit anti-β-actin (bs-0061R, Bioss, China) as a loading control. After three times of washing every 10 min with PBST, membranes were incubated with goat anti-rabbit IgG (Abcam, UK) for 2 h at room temperature. Protein signal was detected using a 3,3′-diaminobenzidine (DAB) electrochemiluminescence system (Beyotime, Jiangsu, China). Optical density was measured using ImageJ software (NIH, Bethesda, MD, USA).

### Data Analysis

Data were expressed as mean ± SD and analyzed using SPSS 22.0 (IBM, Armonk, NY, USA). Intergroup differences in neural function defect score were assessed for significance using one-way ANOVA and the Student-Newman-Keuls test. Intergroup differences in results for immunohistochemistry, Western blotting, or TUNEL assay were assessed for significance using one-way ANOVA and Tukey's *post hoc* test. The value *p* < 0.05 was considered statistically significant.

## Results

### SA Improves Neural Function Score After ICH in Rats

**Figure 3:**
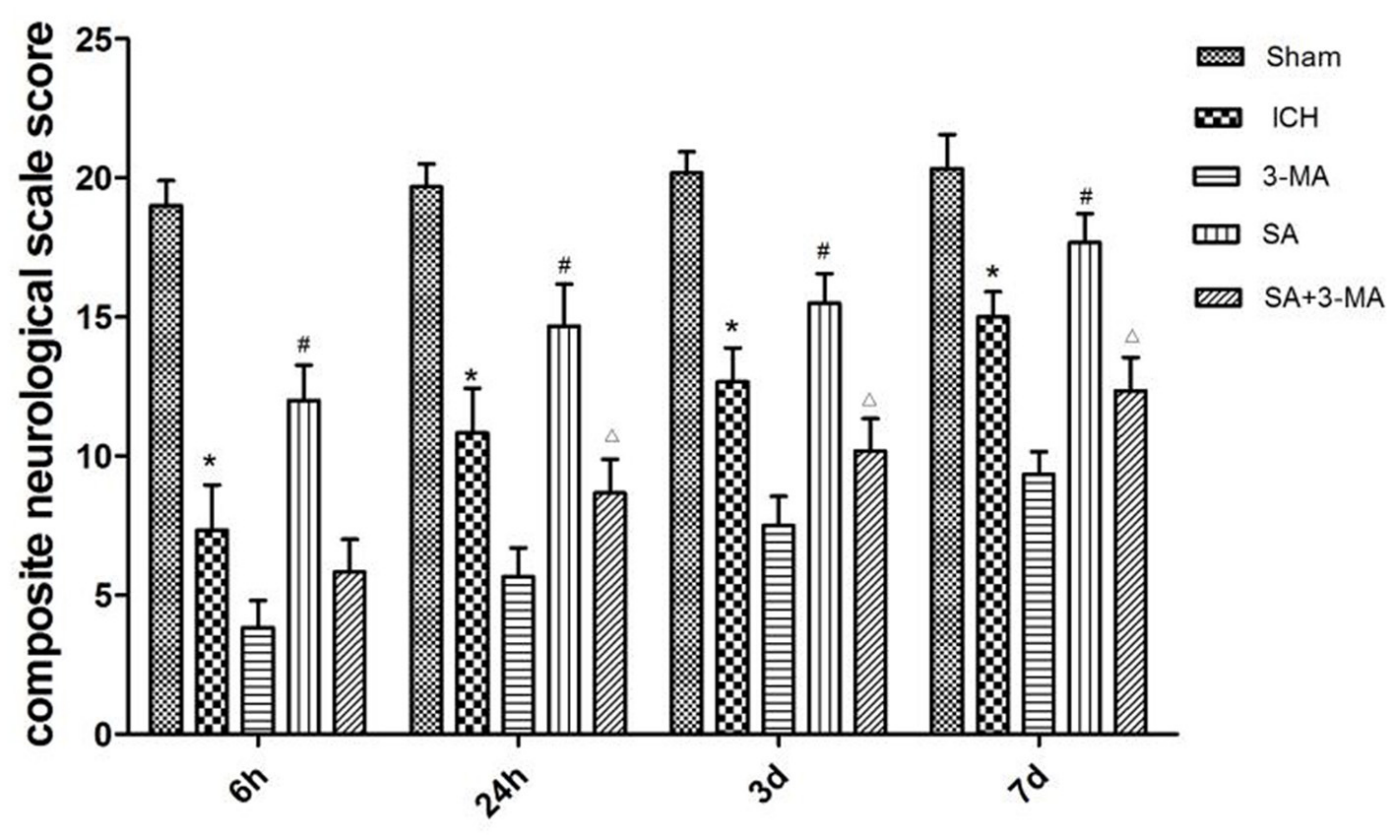
Effect of SA on composite neurological scale score in a rat model of ICH. The results show that SA improves neural function score after ICH in rats. Neuronal function was analyzed in seven behavioral tests. Animals were scored on a scale of 3–21, where lower score indicated more severe neural defect. Data are mean ± SD (*n* = 6). Intergroup differences were analyzed using one-way ANOVA and the Student-Newman-Keuls test. ^*^p < 0.05, vs. sham; ^#^*p* < 0.05, vs. ICH; ^Δ^*p* < 0.01 vs. SA. ICH, intracerebral hemorrhage; SA, scalp acupuncture; 3-MA, 3-methyladenine.

To measure the severity of neural function defects after ICH, we used a composite neurological scale score based on seven physical functions. The sham group showed a similar score across the different time points (Figure 3). The ICH group scored significantly lower than the sham group at all time points. Compared with the ICH group, the SA group scored significantly higher, while the 3-MA group scored significantly lower at all time points. These results suggest that SA can mitigate ICH-related damage to improve neural function.

### SA Can Attenuate ICH-Related Brain Histopathology and Neural Cell Apoptosis

**Figure 4:**
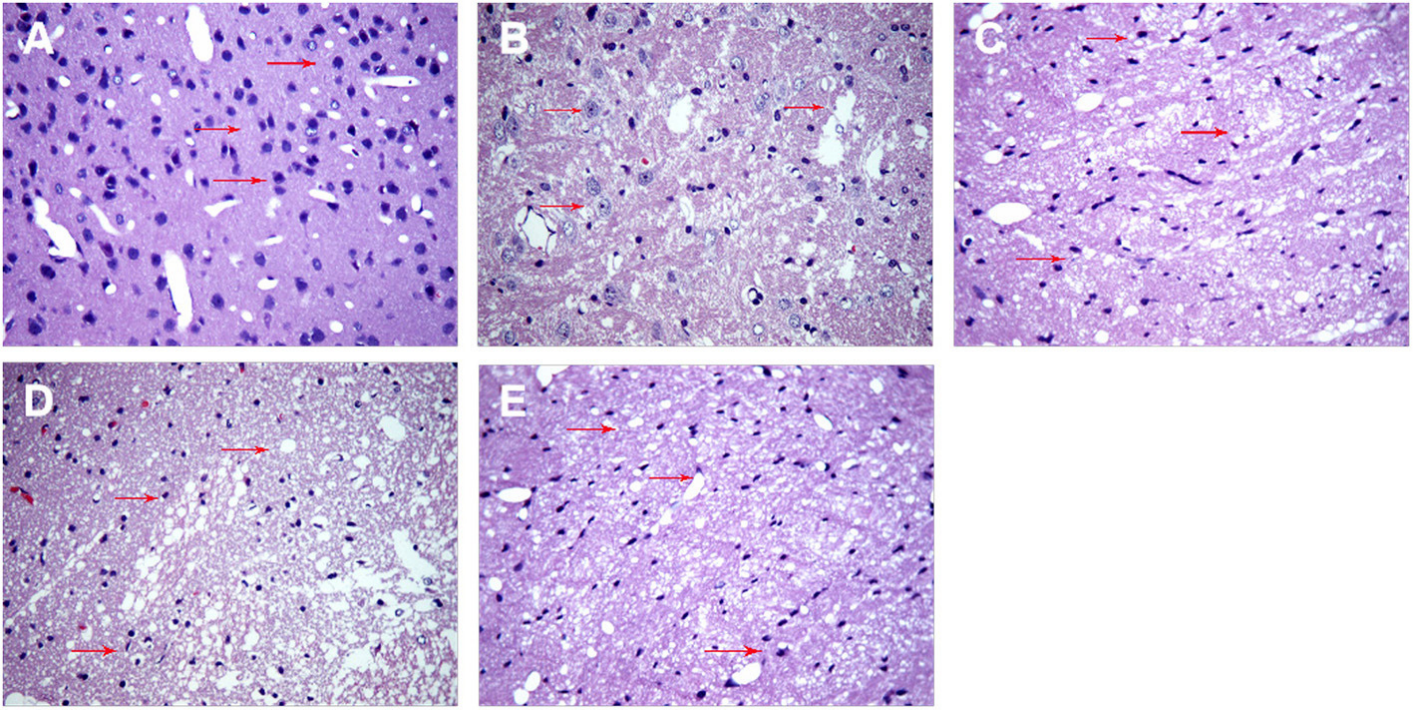
SA mitigates cell injury-edema, inflammatory infiltration in the peri hemorrhagic penumbra of rats with ICH. Brain tissue structure was analyzed at 3 days after sham or ICH surgery by hematoxylin-eosin staining. Representative images are shown from the **(A)** sham, **(B)** ICH, **(C)** 3-MA, **(D)** SA, and **(E)** SA + 3-MA groups. Red arrows indicate inflammatory cells and necrotic cells at the site of injury. Magnification: 400×. ICH, intracerebral hemorrhage; SA, scalp acupuncture; 3-MA, 3-methyladenine.

Next, we examined structural effects of ICH on brain tissue to determine whether SA could attenuate ICH-induced secondary brain damage and apoptosis. At 3 days after sham or ICH surgery, brain tissue was isolated and stained with hematoxylin–eosin (Figure 4) and TUNEL assay (**Figure 6**). Brain tissue from sham-operated animals showed normal morphology and structure, including tight arrangement of cells and uniform chromatin without obvious damage or inflammatory infiltration, the minimum number of apoptotic cells. In contrast, brain tissue from the ICH group showed noticeable damage to neural structures around the hemorrhage, swollen and deformed cells, and vacuoles of various sizes among intercellular space. Tissue from the SA group showed less severe cell injury-edema, inflammatory infiltration, and apoptotic cells than tissue from the ICH group. Conversely, 3-MA exacerbated ICH-induced cell damage, the maximum apoptotic cells number in the 3-MA group. SA+3-MA group showed less severe structural damage than the 3-MA group, suggesting that SA can mitigate the effects of 3-MA. The SA+3-MA group displayed more apoptosis than the ICH group and far more than the SA group. These findings by TUNEL assay were confirmed in Western blotting of brain tissue (**Figure 7**).

### SA Promotes Mitophagy After ICH

**Figure 5:**
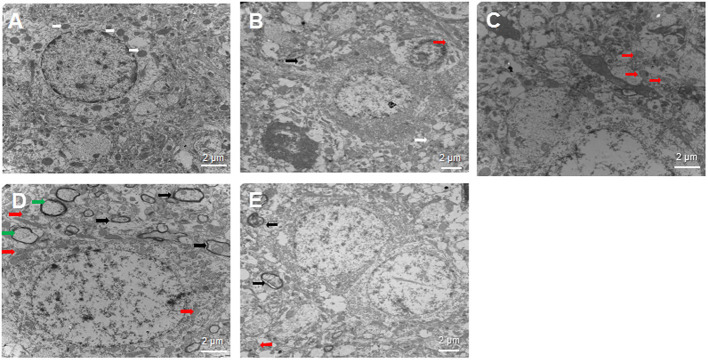
Effect of SA on neural cell ultrastructure after ICH. Autophagy is activated following ICH. Brain samples were isolated from rats at 3 days after sham or ICH surgery and analyzed by transmission electron microscopy. Representative sections are shown from the **(A)** sham, **(B)** ICH, **(C)** 3-MA, **(D)** SA, and **(E)** SA+3-MA groups. Magnification, 10000× **(A, C, D)**, 8000× **(B)** or 7000× **(E)**. White arrows indicate healthy mitochondria; red arrows, damaged mitochondria; black arrows, autophagosome; and green arrows, autolysosomes. Scale bars, 2 μm. ICH, intracerebral hemorrhage.

Intracerebral hemorrhage -induced cellular damage can activate mitophagy as a way to replace damaged mitochondria. We examined neural cells by transmission electron microscopy at 3 days after sham or ICH surgery to determine whether SA can activate mitophagy (Figure 5). In the sham group, the nuclei of neurons were large and round, the nucleoli were clear, and the organelles in the cytoplasm looked normal. Most importantly, mitochondria were abundant and both the inner and outer membranes were clearly visible. In contrast, in the ICH group, the mitochondria were swollen and deformed, the mitochondrial crest was blurred, and it even disappeared completely in some cases. Autolysosomes were observed, suggesting that autophagy was occurring. The 3-MA group showed loose, elongated mitochondria, and a few autophagosomes containing damaged mitochondria. In contrast, the SA group showed less swelling of nerve cells, damage of the cellular and nuclear membrane, and the damage of mitochondrial than that of ICH group. What's more, more autophagosomes and autolysosomes were detected within cells among the SA group. The SA+3-MA group showed more loose mitochondria and autophagosomes than the SA group.

### SA Upregulates Mitophagy Markers in Neural Cells After ICH

**Figure 6:**
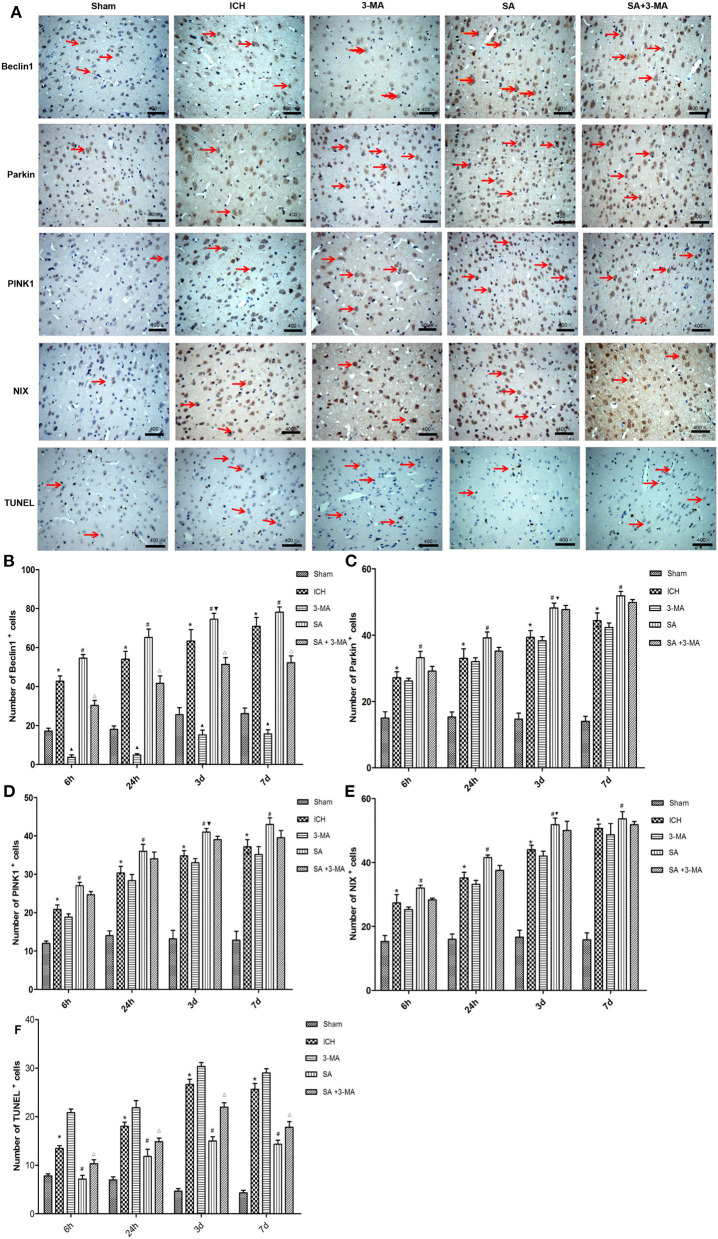
Immunohistochemistry: SA upregulates mitophagy-related proteins and decrease the number of TUNEL positive cells after ICH. **(A)** Immunohistochemistry and TUNEL of brain tissue. Arrows mark positive cells. Magnification, 400×. Scale bars, 50 μm. **(B–F)** Quantitation of numbers of cells positive for Beclin1, PINK1, Parkin, NIX and apoptotic cells in fields of view at 400× magnification. Data are mean ± SD (*n* = 6). Significance was assessed by one-way ANOVA, followed by Tukey's *post hoc* test. ^*^P < 0.05, *vs*. sham; ^#^*P* < 0.05, *vs*. ICH; ^▴^*P* < 0.05, *vs*. ICH; ^Δ^*P* < 0.05, *vs*. SA; ^▾^*P* < 0.05, *vs*. 24h SA group. ICH, intracerebral hemorrhage; SA, scalp acupuncture; 3-MA, 3-methyladenine.

**Figure 7:**
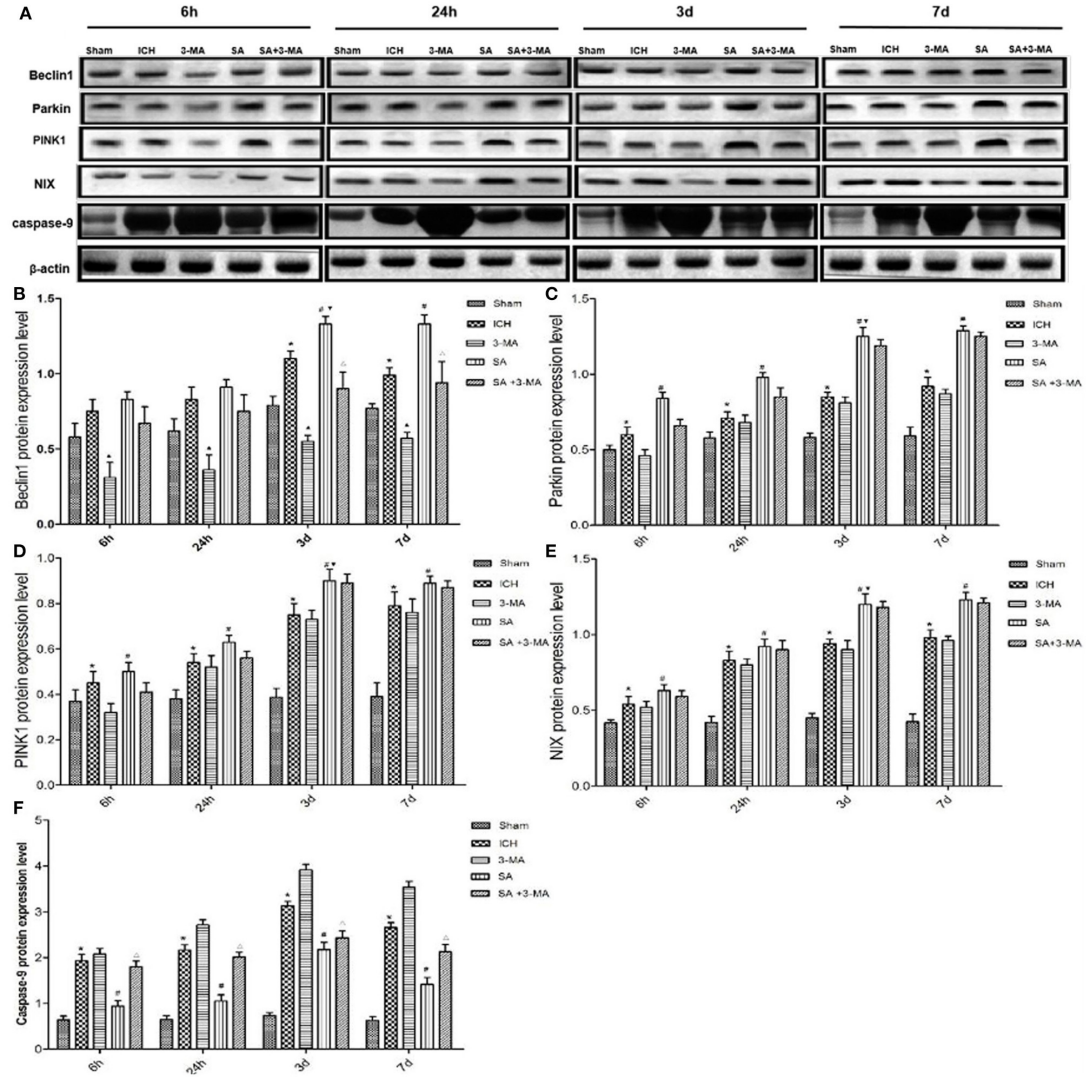
Western blotting: SA upregulates mitophagy-related proteins and decrease the apoptosis-related protein after ICH. **(A)** Western blotting of proteins relevant to mitophagy activation in rat brain tissue after ICH. Protein levels were normalized to levels of β-actin as loading control. **(B–F)** Quantitation of levels of Beclin1, Parkin, PINK1, NIX and Caspase-9. Data are mean ± SD (*n* = 6). Significance was assessed using one-way ANOVA, followed by Tukey's *post hoc* test. ^*^P < 0.05, vs. sham; ^#^*P* < 0.05, *vs*. ICH; ^▴^*P* < 0.05, *vs*. ICH; ^Δ^*P* < 0.05, *vs*. SA; ^▾^*P* < 0.05, *vs*. 24h SA group. ICH, intracerebral hemorrhage; SA, scalp acupuncture; 3-MA, 3-methyladenine.

To confirm mitophagy activation in neural cells after ICH, we examined specific protein markers by immunohistochemistry and Western blot. Brain sections from all the five groups were probed for the presence of PINK1, Parkin, Beclin1, and NIX (Figure 6). Immunohistochemistry revealed significantly higher levels of all proteins in the SA group than the other groups at all time points. Levels of all proteins significantly increased at 3 days and remained stable for up to 7 days after treatment. The 3-MA group showed lower protein levels than the ICH group in the Beclin1, whereas the others tended to show lower levels than the ICH group, although the difference failed to reach statistical significance. These findings by immunohistochemistry were confirmed in Western blotting of brain tissue (Figure 7).

## Discussion

The pathology of ICH is quite complex. Brain injuries caused by hemorrhage can be classified as primary and secondary injuries. Hematoma reduces local tissue blood supply and initiates secondary ischemia reperfusion injury, which involves mitochondrial damage, ultimately leading to neural cell apoptosis through death receptor signaling and endoplasmic reticulum stress signals (Shimada et al., 2012). Mitochondrial impairment induced by the hematoma is a major factor leading to secondary damage. If mitochondria can be preserved through the specific form of autophagy called mitophagy, this secondary damage may be ameliorated. The inflammatory reaction and coagulation cascade caused by hematoma can lead to edema of the surrounding brain tissue, which causes the more serious and lasting damage, and has a negative impact on the prognosis of intracerebral hemorrhage. Our previous studies have shown that SA can reduce the water content of brain tissue in rats with ICH to alleviate the secondary injury of brain edema. Further study found that SA could reduce inflammatory injury and brain edema by regulating the Mincle/Syk pathway (Liu et al., 2018b). SA could reduce neuronal death and inflammation and alleviate brain edema after intracerebral hemorrhage by downregulating miR-23a-3p (Kong et al., 2021). Research has confirmed that acupuncture may enhance mitochondrial respiratory chain enzyme activity and improve mitochondrial dysfunction (Zhang et al., 2014). Previous studies in our team have indicated that SA may preserve neuronal structure and neural function after ICH through inhibition of cell apoptosis by Sonic hedgehog pathway and activation of autophagy (Zhang et al., 2018; Liu et al., 2019). In this work, we induced ICH in male rats through the injection of autologous blood, and then we examined the possibility that SA protected against neuronal apoptosis and brain tissue damage through eliminating damaged mitochondria by mitophagy pathway. The mechanism elucidated in this work might be conducive to providing a new direction for the clinical application and basic research of SA treatment for ICH.

In this study, we found that SA could improve neural function score, reduce the number of apoptotic cells, alleviate mitochondrial damage, and significantly promote expression of mitophagy-related proteins in our rat model of ICH. Using transmission electron microscopy, immunohistochemistry, and Western blotting, we found that not only ICH but also SA activated mitophagy through the PI3KIII, PINK1/Parkin, and NIX pathways in brain tissue. More importantly, the extent of increased mitophagy was higher in SA group than in ICH group, while mitochondrial damage, neuronal apoptosis, and brain injury in ICH group were significantly higher than the SA group. Accordingly, we speculated that SA might attenuate brain damage after ICH through enhanced mitophagy, a consequence of reduced apoptosis. Further, we applied 3-MA to inhibit autophagy by blocking autophagosome formation *via* the inhibition of class III PI3K (Castino et al., 2010). Compared with SA group, we found that the score, structural damage, and expression of mitophagy proteins in the SA+3-MA group were decreased, especially of Beclin1, but the level of Caspase-9 was increased in our rat model of ICH. It was found that 3-MA exacerbated hypoxic-ischemic brain damage in neonatal rats by inhibiting mitophagy, resulting in infarct expansion (Liu et al., 2017). Together, we provided evidence that SA might prevent the brain tissue impairment from ICH injury through enhanced mitophagy, thus reducing apoptosis.

Physiological activation of autophagy can eliminate the accumulation of damaged organelles and abnormal proteins, inhibiting the expansion of harmful signals and the death of neurons (Liu et al., 2010). Autophagy promotes cell survival under stress conditions such as starvation, hypoxia, and infection (He et al., 2012; Choi et al., 2013; Green and Levine, 2014). Studies have shown that enhancing autophagy plays a protective role in nervous system diseases. In a model of subarachnoid hemorrhage in rats, activation of autophagic pathways could reduce early brain injury (Jing et al., 2012). Research reported that electroacupuncture might strengthen autophagy *via* mTOR signaling pathway, reducing the early impairment in cerebral ischemia rats (Wu et al., 2016). It is consistent with our current results.

Whether autophagy exerts profitable or detrimental effection remains controversial. It is suggested that activation of autophagy could aggravate brain injury in ICH model, which may be relevant to regulation of NF-kB pathway, thus promoting inflammatory response and apoptosis (Shen et al., 2016). The reasons for the contradiction between the results above and that of our work may be involved in different methods of modeling animal species and intervention methods. Whether there exists appropriate, overactivated, or defective autophagy in the course of this disease deserves further investigation.

Scalp penetration acupuncture therapy for ICH has been proved to be clinically effective (Zheng et al., 2011). It can ameliorate patient neurological defects, make it easier to obtain the sense of “Deqi,” and reduce the effect of pain, achieving a better prognosis (Wang et al., 2016). The inhibitory effects of acupuncture on apoptosis in brain tissue after ICH have been confirmed. Previous studies have shown that acupuncture can inhibit the apoptosis of brain tissue cells in ICH model rats by upregulating the level of Bcl-2 mRNA and downregulating the level of Bax mRNA (Li et al., 2017). Acupuncture can reduce apoptosis through endoplasmic reticulum stress (Sun et al., 2020), oxidative stress (Li et al., 2018), and PI3K/Akt signaling (Wang et al., 2019). Both acupuncture and electroacupuncture are considered as nondrug treatments for ICH. Electroacupuncture can also increase Bcl-2 and reduce Caspase-3, Bax, and p53 in the brain tissue of rats with ICH (Zhu et al., 2017; Guan et al., 2021). However, electroacupuncture and acupuncture are two different stimulation methods. Electroacupuncture relies on electric current to stimulate tissue. Acupuncture we use achieved the purpose of stimulation through mechanical actions such as lifting, inserting, twisting, and turning. The effect of electroacupuncture on organisms cannot be completely equal to that of acupuncture. In this study, we focused on the selective autophagy pathway-mitophagy. The results showed that acupuncture could reduce the apoptosis of brain cells by enhancing mitophagy, and then improve neurological function. This finding has rarely been reported in previous studies. However, this study did not explore the mechanism regulating autophagy and apoptosis by SA. Our study provided some insights so far into the relationship between mitophagy and post-ICH neural repair, which might implicate the PI3KIII, PINK1-Parkin, and NIX signaling pathways. These pathways are unlikely to be the only ones involved after ICH, since mitophagy can also occur in mice lacking Parkin and NIX (Yuan et al., 2017). In future, we will explore the role of other mitochondrial pathways in ICH model and realize the mechanism of SA for mitophagy enhancement and apoptosis attenuation.

## Data Availability Statement

The raw data supporting the conclusions of this article will be made available by the authors, without undue reservation.

## Ethics Statement

The animal study was reviewed and approved by Animal Experiment Center of Heilongjiang University of Chinese Medicine.

## Author Contributions

PL, WZ, and X-PY: designed the study. PL: wrote the manuscript. X-YY and X-HD: conducted most of the experiments and collected the data. MN, QC, WT, YK, X-YL, and R-QG participated in the discussion and helped with the experiment. All authors have read and approved the final.

## Funding

This study was funded by the National Natural Science Foundation of China (No. 81774416). Heilongjiang University of Chinese Medicine Graduate Innovation Fund Project (No. 2017yjscx005).

## Conflict of Interest

The authors declare that the research was conducted in the absence of any commercial or financial relationships that could be construed as a potential conflict of interest.

## Publisher's Note

All claims expressed in this article are solely those of the authors and do not necessarily represent those of their affiliated organizations, or those of the publisher, the editors and the reviewers. Any product that may be evaluated in this article, or claim that may be made by its manufacturer, is not guaranteed or endorsed by the publisher.
